# Metacognition, Hardiness, and Grit as Resilience Factors in Unmanned Aerial Systems (UAS) Operations: A Simulation Study

**DOI:** 10.3389/fpsyg.2019.00640

**Published:** 2019-03-26

**Authors:** Gerald Matthews, April Rose Panganiban, Adrian Wells, Ryan W. Wohleber, Lauren E. Reinerman-Jones

**Affiliations:** ^1^Institute for Simulation and Training, University of Central Florida, Orlando, FL, United States; ^2^Air Force Research Laboratory, Dayton, OH, United States; ^3^Division of Psychology and Mental Health, School of Health Sciences, The University of Manchester, Manchester, United Kingdom; ^4^Greater Manchester Mental Health NHS Foundation Trust, Prestwich, United Kingdom

**Keywords:** metacognition, worry, grit, resilience, stress, psychophysiology, Unmanned Aerial Systems, workload

## Abstract

Operators of Unmanned Aerial Systems (UAS) face a variety of stress factors resulting from both the cognitive demands of the work and its broader social context. Dysfunctional metacognitions including those concerning worry may increase stress vulnerability, whereas personality traits including hardiness and grit may confer resilience. The present study utilized a simulation of UAS operation requiring control of multiple vehicles. Two stressors were manipulated independently in a within-subjects design: cognitive demands and negative evaluative feedback. Stress response was assessed using both subjective measures and a suite of psychophysiological sensors, including the electroencephalogram (EEG), electrocardiogram (ECG), and hemodynamic sensors. Both stress manipulations elevated subjective distress and elicited greater high-frequency activity in the EEG. However, predictors of stress response varied across the two stressors. The Anxious Thoughts Inventory (AnTI: Wells, 1994) was generally associated with higher state worry in both control and stressor conditions. It also predicted stress reactivity indexed by EEG and worry responses in the negative feedback condition. Measures of hardiness and grit were associated with somewhat different patterns of stress response. In addition, within the negative feedback condition, the AnTI meta-worry scale moderated relationships between state worry and objective performance and psychophysiological outcome measures. Under high state worry, AnTI meta-worry was associated with lower frontal oxygen saturation, but higher spectral power in high-frequency EEG bands. High meta-worry may block adaptive compensatory effort otherwise associated with worry. Findings support both the metacognitive theory of anxiety and negative emotions (Wells and Matthews, 2015), and the Trait-Stressor-Outcome (TSO: Matthews et al., 2017a) framework for resilience.

## Introduction

Individual differences in resilience and stress vulnerability have profound personal consequences for life outcomes such as career success, personal relationship quality, and mental health. Recent work has demonstrated the complexity of resilience, which depends on multiple personality traits whose influence on stress outcomes varies across different demanding contexts (Matthews et al., 2017a). The present study explores the contribution to resilience and stress vulnerability of worry traits including meta-worry, i.e., metacognitive worry about worry itself (Wells, 1994, 2005). A simulation of Unmanned Aerial System (UAS) control provided a testbed for manipulating stress and assessing multiple components of stress response. The overall aim was to examine how worry and additional traits for resilience predicted stress response within the frameworks provided by the metacognitive theory of maladaptive emotions (Wells and Matthews, 1996, 2015) and multifactorial resilience theory (Matthews et al., 2017a).

### Worry, Metacognition, and Stress

Personality traits for emotional vulnerability and resilience can be broadly divided into maladaptive traits that amplify harmful impacts of stressors and adaptive traits that support effective coping. Beyond broad traits such as neuroticism, theoretical considerations suggest a focus on dispositional worry and metacognition. Specifically, the Self-Regulatory Executive Function (S-REF) theory (Wells and Matthews, 1996, 2015) defines a Cognitive Attentional Syndrome (CAS) associated with perseverative worry, rumination and threat-monitoring that interferes with task-directed attention and causes psychological dysfunction. The CAS is typically triggered by an external threat or an intrusive thought, but persistence of the syndrome results from metacognitions that maintain attention on negative self-referent thoughts. For example, the person may believe that intrusive thoughts are important, that worrying about them will resolve personal concerns, or that thoughts are uncontrollable (Wells, 2000). The impact of metacognitions in the S-REF model is multifaceted, potentially impacting motivation to regulate cognition and utilize effort, choice of response strategy, and the threat assigned to cognitive processes themselves.

Dispositionally worry-prone individuals are vulnerable to the CAS and states of worry in performance settings (Matthews and Funke, 2006). Dispositional worry and allied constructs such as rumination are also risk factors for both subclinical stress reactions to life events and emotional disorders (Kircanski et al., 2017; Ryum et al., 2017). However, dispositional worry is itself a complex construct, that may variously include specific personal concerns such as health and social status (Wells, 1994), maladaptive styles of stress processing such as excessive threat appraisal and avoidance coping (Borkovec et al., 2004; Matthews and Funke, 2006), and metacognitive factors, such as beliefs about worry as specified by S-REF theory (Wells and Matthews, 2015).

Evidence from both experimental and correlational studies demonstrates the role of metacognitions in acute stress in non-clinical samples. Palmier-Claus et al. (2011) used disturbing images as a stressor. They found that the negative affect response was stronger in individuals with negative metacognitive beliefs referring to the importance of controlling one’s thoughts, and the uncontrollability of thoughts. Capobianco et al. (2018b) induced metacognitions directly with a fake EEG manipulation that lead participants to believe their negative thoughts would trigger a burst of aversive noise. The manipulation amplified and prolonged the negative emotional response to a subsequent stressor (the Trier Social Stress Test). A further experimental study utilizing the Trier test (Capobianco et al., 2018a) showed that a group in whom worry was induced experimentally showed elevated negative affect immediately following the stressor exposure.

In correlational studies, dysfunctional metacognitions have been associated with test anxiety and maladaptive coping (Matthews et al., 1999), perceived stress symptoms (Roussis and Wells, 2006; Spada et al., 2008), state anxiety (Spada et al., 2010), and anxiety and depression when life events are controlled (Yılmaz et al., 2011). Because these various stress responses are likely to distract from attention to tasks, it is expected that the negative metacognitions that drive them will be maladaptive in the performance context. Whilst there is a large body of research supporting central predictions of the S-REF model of vulnerability (Wells, 2013; Wells and Matthews, 2015), little is known about factors associated with the CAS and metacognitions that enhance resilience.

### A Multifactorial Perspective on Resilience: The TSO Framework

The current study focuses on stress response during performance of a multi-component cognitive task. A basic challenge in identifying the role of metacognition in this context is the complexity of individual differences in stress response. Resilience traits additional to metacognitive factors may also influence response. Furthermore, the nature of the stressor may moderate the relationship between traits for resilience and stress outcomes. Findings may depend too on the stress outcome measure examined. For example, psychophysiological measures can pick up stress responses of which the person is not consciously aware (Verkuil et al., 2010). Matthews et al. (2017a) proposed a Trait-Stressor-Outcome (TSO) framework for specifying dispositional individual differences in stress response across different contexts. It emphasizes that the traits that predict stress reactivity vary from stressor to stressor, and influence different stress outcomes. From the TSO perspective, we may ask which stressors elicit differential responding in individuals differing in metacognition, which outcome measures demonstrate differential response, and how the role of metacognition compares to other relevant resilience traits.

The present study investigated the stress of operating multiple UASs, aerial vehicles controlled remotely for purposes including reconnaissance and surveillance. Current military and civilian operations typically involve a two- or three-person team controlling the vehicle; in the future a single operator will control multiple vehicles with assistance from automation (Calhoun and Draper, 2015; Wohleber et al., 2018). Stressors include the cognitive challenges of managing complex interfaces, variable workload, social evaluation, and long workshifts (Tvaryanas et al., 2006; Paullin et al., 2011). Some of these stressors are more likely to elicit the CAS than others. Social-evaluative stress commonly elicits both worry (Zeidner, 1998) and physiological stress response (Dickerson and Kemeny, 2004). In the military training context, stress response is accentuated when trainees feel that their performance is being judged by peers and instructors and they receive critical feedback (Carroll et al., 2014). By contrast, high cognitive workloads are also stressful but they may direct attention outward to manage high volumes of external task stimuli, limiting the potential onset of the CAS.

Performance stress is expressed in various ways, through subjective experience, changes in neural functioning, and objective performance impairment. Subjective states experienced in performance environments may be assessed using the Dundee Stress State Questionnaire (DSSQ: Matthews et al., 2002, 2013). It identifies 11 primary affective, motivational and cognitive dimensions that define higher-order factors of task engagement, distress, and worry. The worry state combines self-focused attention, low performance self-esteem, and intrusive thoughts about the task and personal concerns. Matthews (2016) reviewed studies showing that different task stressors elicit different patterns of response across the dimensions, that reflect the different appraisals and coping strategies that shape each state dimension. Acute stress response is often identified with sympathetic arousal but studies of stress elicited by high-workload tasks reveal a more complex picture. Matthews et al. (2015) recorded electrocardiac, electroencephalographic, and hemodynamic responses to multiple tasks, and found that responses from different physiological systems dissociated, implying that multiple brain systems may underpin response to task stressors. Multivariate assessment is important because different responses may have differing functional significance. For example, in a simulation of unmanned ground vehicle operation, Matthews et al. (2017b) found that subjective and physiological measures contributed independently to performance prediction; high distress, low heart rate variability, and high frequency EEG were all associated with performance impairment.

The TSO framework assumes that multiple traits may moderate stress response, depending on the context. Traits for resilience refer to focus on positive qualities supporting coping, whereas stress vulnerability traits define qualities such as worrying that are detrimental to coping. Broad trait models typically characterize positive and negativity emotionality dimensions as largely independent (Watson, 2000), but it is unclear whether resilience and vulnerability traits can be neatly partitioned into two separate categories; for example, dysfunctional metacognition may undermine the task-directed motivations that support resilience. According to TSO, traits adaptive for stressful performance settings should be those that maintain attentional focus, task-directed effort and problem-focused coping. Multiple traits are potentially relevant, but here we focus on hardiness (Bartone et al., 1989; Escolas et al., 2013), and grit (Duckworth and Quinn, 2009). Such traits have cognitive, motivational, and emotional aspects, but their relationship to metacognition and the CAS of S-REF theory (e.g., worry) is unknown.

The construct of hardiness as a general trait for resilience emerged from studies of personality traits that might buffer the health impacts of life stressors (Kobasa, 1982). Scales for hardiness (e.g., Bartone et al., 1989) have been widely utilized in studies of stress in organizational, military and other contexts, generally confirming that the trait enhances resilience and performance under stress (Maddi et al., 2012). A meta-analysis (Eschleman et al., 2010) confirmed that hardiness is substantially correlated with various measures of higher well-being and lower stress, including lower scores on measures of depression and traumatic stress. Hardiness was also associated with adaptive cognitive stress processes such as preferences for problem-focused and approach coping over avoidance and emotion-focused coping. Hardiness also correlates with more constructive appraisals (Cash and Gardner, 2011). The role of metacognitive style in hardiness has been overlooked. However, because the adaptive pattern of coping and appraisal associated with the trait tends to mitigate against development and maintenance of the CAS (Wells and Matthews, 2015), it is hypothesized that hardiness will be negatively associated with maladaptive metacognition.

Definitions of grit focus on long-term persistence and maintenance of motivation during adversity (Duckworth and Quinn, 2009), but this trait may also influence acute stress response to task performance challenges. Grit correlates positively with wellbeing and mental health, and negatively with stress and symptoms of depression (Goodman et al., 2017; Sharkey et al., 2017; Kannangara et al., 2018), although the literature is not fully consistent (Kannangara et al., 2018). Grit also correlates with cognitive and self-regulative processes that may confer resilience including positive control beliefs (Goodman et al., 2017), self-efficacy (Muenks et al., 2018), and self-control (Duckworth and Gross, 2014). It is also associated with lower levels of brooding and reflective rumination (White et al., 2017). In addition, studies of grit in the academic context show relationships with processes supporting self-regulated learning including adaptive metacognitive strategies for planning, monitoring and regulating the learning process (Wolters and Hussain, 2015). Thus, high-grit individuals should be more effective at self-regulation when required to perform a stressful cognitive task. From a theoretical standpoint, grit is associated with positive attitudes despite setbacks and failure (Lucas et al., 2015), and with low levels of ruminative processes (White et al., 2017). These characteristics should act against prolonged CAS activation in stressful task environments.

### The Present Study

There has been rather little research on the relationship between dispositional worry, metacognition, resilience, and stress responses in complex, demanding performance environments. This lack of evidence represents a limitation of both CAS and TSO models. In the current multi-UAS control task, the participant must guide vehicles to target locations and photograph them while monitoring for vehicle health and avoiding areas of danger. Panganiban and Matthews (2014) developed and validated two stress manipulations, one that increased cognitive demand and one that delivered negative feedback about performance. We considered that negative feedback was more likely than high cognitive demand to activate the CAS, because it involved direct personal criticism.

In the present study, a within-subjects design was used. All participants performed under both stressors, as well as in two control conditions, one prior to each stressor (four conditions in total). We aimed to test whether traits for stress vulnerability (i.e., those predisposing activation of the CAS) and resilience predicted physiological and subjective responses, utilizing a suite of sensors previously applied across a range of demanding task environments (Matthews et al., 2015; Reinerman-Jones et al., 2016). We administered the Anxious Thoughts Inventory (AnTI: Wells, 1994), which assesses traits related both to specific worry concerns (social and health) and to meta-worry, along with scales for two adaptive resilience constructs, hardiness (Bartone et al., 1989) and grit (Duckworth and Quinn, 2009).

Stress responses in demanding performance environments change dynamically throughout the test session (Matthews and Campbell, 2010). People differ in anticipatory stress and worry before exposure to stressors (Brosschot et al., 2006); a study of medical students (O’Carroll and Fisher, 2013) found that trait anxiety and metacognitive factors predicted anxiety immediately prior to a clinical examination. Thus, evaluating stress reactivity requires control for individual differences in stress at baseline. In this study, we analyzed both baseline and reactivity data. Associations between resilience traits and subjective states at baseline identifies factors associated with stress state in the absence of substantial overt demands. We also evaluated individual differences in reactivity, by testing for associations between traits and stress response with baseline levels of stress controlled. Subjective and physiological responses were analyzed to test for specificity of response; i.e., some individuals might show strong responses to negative evaluation but not cognitive overload, and vice versa. Having identified individual differences in stress reactivity to negative feedback, we then ran a further analysis to test whether dispositional meta-worry moderated associations between worry and objective outcomes as predicted by the CAS model. The specific research issues addressed were as follows:

#### Stress Profiles of Cognitive Demand and Feedback Stressors

We expected that both stress manipulations would elevate subjective distress (Panganiban and Matthews, 2014) and psychophysiological stress indices including high-frequency EEG activity (Reinerman-Jones et al., 2016). However, we also anticipated qualitative differences in response associated with each stressor, including higher workload with cognitive demand and higher state worry with negative feedback.

#### Associations Between Traits and Stress States: Baseline and Control Conditions

Metacognitive factors correlate with perceived stress in the absence of an overt stressor (Spada et al., 2008), and traits for worry and metacognition are associated with anticipatory anxiety (O’Carroll and Fisher, 2013). Thus, we hypothesized that the AnTI traits would be associated with elevated DSSQ distress and worry, as well as psychophysiological stress measures. We also anticipated negative associations between AnTI traits and hardiness and grit, as well as correlations between these traits and higher task engagement, lower distress, and lower worry.

#### Worry and Resilience Traits and Reactivity to Stressors

We tested whether traits would predict stress response over and above any associations evident in the control conditions. To do this, we computed measures of stress reactivity specific to each stressor. We expected that the AnTI would predict subjective and physiological responses to negative feedback more strongly than responses to cognitive demand, because feedback is more likely to activate the CAS due to its higher self-relevance. Accounts of hardiness and grit do not clearly link these qualities to specific stressors so their associations with reactivity were investigated on an exploratory basis.

#### Metacognition and the Functional Significance of Worry

Worry states are broadly if modestly detrimental to performance (Zeidner, 1998; Matthews and Funke, 2006), but recent work has also identified potential functional advantages of worry including motivating problem-solving and coping efforts (Sweeny and Dooley, 2017). We can infer from the S-REF theory (Wells and Matthews, 2015) that relationships between worry and adaptive outcomes may be moderated by metacognitive style. Specifically, individuals high in meta-worry are likely to react to the awareness of worry by re-directing attention and effort from task performance to processing and regulating the worry state, whereas those low in meta-worry are more likely to use worry as a spur to increase task-directed effort. This hypothesis was tested against objective measures of performance and psychophysiological response in the negative feedback stressor condition, in which CAS activation was most likely.

## Materials and Methods

### Participants

Participants were 68 undergraduate students (31 women, 37 men, *M*_age_: 19.3 years) at the University of Central Florida. They received course credit for participation. Participants were excluded if they reported current or recent treatment for any emotional disorder, eating disorder, schizophrenia or other psychosis, stress or any related emotional condition. Those currently taking psychoactive medications were also excluded.

### Subjective Measures

#### Anxious Thoughts Inventory (AnTI: Wells, 1994)

This questionnaire includes 22 items answered on 4-point response scales. It includes subscales for social worry (e.g., “I worry about my appearance”), health worry (e.g., “I have thoughts about becoming seriously ill”), and meta-worry (e.g., “I have difficulty clearing my mind of repetitive thoughts”). Subscale alpha coefficients quoted by Wells (1994) ranged from 0.75 to 0.84.

#### Hardiness Scale (Bartone et al., 1989)

This measure of resilience has 30 items, answered on 4-point response scales. The subscales are commitment (e.g., “Most days, life is really interesting and exciting to me,” challenge (e.g., “I like it when things are uncertain or unpredictable”), and control (e.g., “When I make plans, I’m certain I can make them work”). Bartone et al. (1989) reported an alpha of 0.83 for total hardiness, and subscale alphas ranging from 0.62 to 0.82.

#### Short Grit Scale (Duckworth and Quinn, 2009)

This questionnaire includes 12 items, answered on 5-point response scales, which assess capacity to sustain effort and interest in demanding activities (e.g., “Setbacks don’t discourage me”). Scale alphas in four samples ranged from 0.73 to 0.83.

#### Short Dundee Stress State Questionnaire (DSSQ: Matthews et al., 2013)

The short, 21-item version of the DSSQ assesses subjective state responses related to task engagement (e.g., “I was determined to succeed”), distress (e.g., “I felt tense”), and worry (e.g., “I reflected about myself”). Items are answered on 4-point scales. Scale alphas range from 0.78 to 0.83 (Matthews et al., 2013).

#### NASA Task Load Index (NASA-TLX: Hart and Staveland, 1988)

This workload measure requires the respondent to use 0–100 scales to rate 6 sources of task load (mental demand, physical demand, temporal demand, effort, frustration, performance). Overall workload is calculated as an average of ratings, with performance reverse scored. The scale authors reported a test-retest reliability of 0.83.

### Psychophysiological Measures

A suite of sensors used in previous studies recorded multiple psychophysiological responses. Brief descriptions are given here: see previous reports for further detail (Barber et al., 2011; Matthews et al., 2015). Multiple responses were recorded simultaneously during an initial baseline period and throughout task performance.

#### Electroencephalogram (EEG)

The ABM B-Alert X10 system assessed nine channels of EEG. Following filtering and artifact removal, spectral power was averaged across three frontal sites for theta (4–8 Hz), alpha (9–13 Hz), beta (14–30 Hz), and gamma (30–100 Hz) bandwidths. EEG data were analyzed as percent change from baseline.

#### Electrocardiogram (ECG)

The ABM System B-Alert X10 system also recorded ECG. Mean Inter-Beat Interval (IBI) and Heart Rate Variability (HRV) were recorded. IBI was analyzed as percent change from baseline for each task condition. HRV was calculated as the SD of all beats (measured in ms) during each condition.

#### Functional Near-Infrared Spectroscopy (fNIR)

Hemodynamic changes in the left and right hemispheres of the prefrontal cortex were measured using Somanetics’ INVOS Cerebral/Somatic Oximeter. The fNIR method analyzes the spectral absorption of NIR light by brain tissue. Regional oxygen saturation (rSO^2^) during each condition was calculated as the percent change from baseline.

#### Transcranial Doppler Sonography (TCD)

Cerebral blood flow velocity (CBFV) in the left and right hemisphere middle cerebral arteries was measured using Spencer Technologies’ ST3 Digital Transcranial Doppler system. The system transceiver emits ultrasound pulses that are reflected back to the sensor from the moving blood cells; velocity is calculated from analysis of the Doppler shift in frequency. CBFV was calculated as the percent change from baseline.

### Apparatus

**Figure 1:**
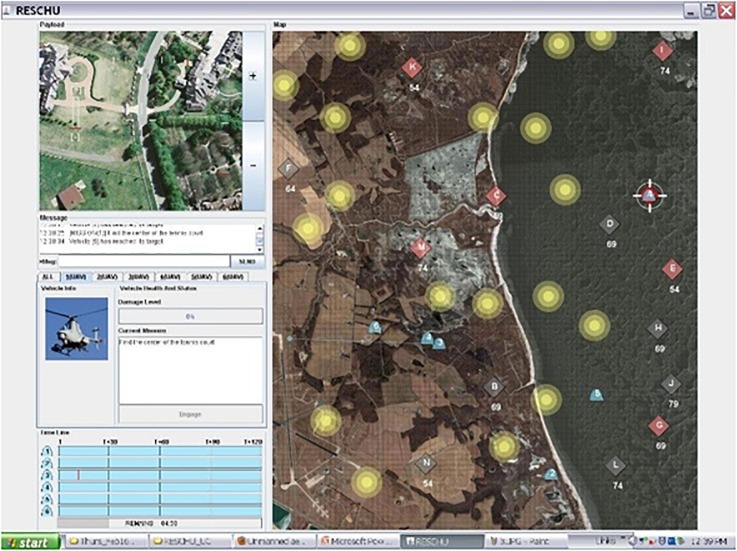
RESCHU simulator. The payload window for search tasks is located on the top left. The message window is below the payload window, and below that is a vehicle information display. The map display shows targets (red and gray), hazards (yellow), and UASs (blue).

We used the Java-based “Research Environment for Supervisory Control of Heterogeneous Unmanned Vehicles” (RESCHU) multi-UAV simulator developed by the Human and Automation Lab at the Massachusetts Institute for Technology (Boussemart and Cummings, 2008). Full details are provided by Panganiban (2013). In brief, RESCHU simulates complex dynamic supervisory control. A single operator controls multiple UASs performing surveillance missions, using the mouse to control the vehicles via a point-and-click interface. The display includes multiple windows as shown in Figure 1. The aim was to assign UASs to searchable targets represented by red and gray diamond symbols on a map display. Participants visually identified key objects on arrival of the UAS at the target site. Each UAS was identified by a number. To perform the task, the participant first allocated a UAS to a given target, allocating odd-numbered UASs to red targets and even-numbered UASs gray targets. The participant then used the mouse to define waypoints along a path to the target, in order to avoid hazardous regions, represented by yellow circles. If a UAS entered such a region it took damage. When the UAS arrived at the target, the participant was informed in the message window. The participant accessed a “payload window” that displayed a camera view of the ground below. The message window specified a specific object to locate, such as “yellow car” or “a building with a blue roof.” The participant used the mouse to control the camera view and to zoom in and out as necessary to locate the object. The task is made more difficult by the expiration of targets and the disappearance and reappearance of hazards. Target areas and hazard areas have countdown timers and each moves to a new position on the map once its timer reaches zero. The task is considered to require multiple cognitive capabilities including planning, visual scanning, visual memory, allocation of attention, and multi-tasking which together support integrated executive functioning in a complex and dynamic task environment (Ratwani et al., 2010).

Stress manipulations were similar to those used by Panganiban and Matthews (2014). For lower-stress control trials, participants controlled two UASs. Fourteen targets and nine hazards were present on the screen. Targets expired after 60 s and hazards relocated every 5 s. In the *negative feedback* stressor condition, the same task configuration was used, but scripted feedback referring to participants’ performance was provided in the mission window every 30 s. Approximately two-thirds of the feedback statements were negative (e.g., “You are not meeting expectations”); the remainder were neutral (“You are performing adequately”). Messages were presented in a pseudo-random sequence unrelated to actual performance. This manipulation was expected to activate the CAS in vulnerable individuals. In the *cognitive demand* stressor condition, cognitive demands were increased by increasing the number of UASs, the numbers of targets and hazards, and decreasing the time for which each target was available. In this condition, participants controlled six UASs, and with 18 targets and 14 hazards consistently present on the screen. Targets expired after 45 s, hazards after 5 s. Measures of performance effectiveness were (1) the command ratio, the number of targets engaged divided by the number of targets assigned, and (2) search accuracy, the number of objects located divided by the number of targets engaged. We also assessed (3) waypoints added, the total number of waypoints set in routing vehicles to targets.

### Procedure

Following an informed consent interview, participants completed questionnaires including the AnTI, Hardiness and Grit scales, and pre-task DSSQ. The physiological sensors were then attached and data recording quality was verified. Participants watched a blank screen for 5 min during which baseline physiological measures were secured. Participants then received training on the task. They viewed a Powerpoint slideshow which explained the nature of the task and then practiced on the lower cognitive demand version of the task. Performance was monitored by the experimenter to ensure participant competence was sufficient to move onto the main part of the task. Participants then performed a sequence of four trials in one of two orders; either control – negative feedback – control – high demand or control – high demand – control – negative feedback. Thus, each stressor was preceded by its own control condition. Order was counterbalanced across participants. Stressor trials were 10 min in duration; control trials were 5 min. After each trial, the participant completed the NASA-TLX and a post-task DSSQ. Finally, physiological sensors were removed and participants were debriefed.

## Results

The study provided an extensive data set. Thus, analyses were targeted to address the four research issues previously identified, and they are presented as follows. First, we verified that the two stressors were effective in eliciting stress responses, and we ran ANOVAs to test whether they elicited different patterns of stress response. Second, we computed correlations between the various traits and stress states in relatively undemanding conditions, i.e., at baseline and in control conditions. This analysis tested whether the AnTI correlates with stress in the absence of an overt stressor. Third, we computed correlations between traits and the stress reactivity measures for the cognitive demand and negative feedback conditions, testing whether the AnTI specifically predicts stress response to feedback, as hypothesized. Fourth, we focused in on the role of meta-worry as a moderator of responses to negative feedback. We used a regression approach to test for interactions between AnTI meta-worry and subjective worry state in predicting objective performance and physiological outcomes, testing for whether meta-worry controls whether or not worry states are maladaptive.

### Stress Profiles of Cognitive Demand and Negative Feedback Stressors

Dependent stress response measures were the three DSSQ scales, NASA-TLX workload, and the psychophysiological measures from EEG, ECG, fNIR and TCD. A 2 × 2 (stress level × stress type) repeated measures ANOVA was run for each one. A significant main effect of stress level, with no interaction, implies that both stressors influenced the response measure. A significant interaction indicates a differential effect of stressors on the measure. The significant effects in this analysis are summarized in Table 1 (full ANOVA tables are available from the authors). There were no significant effects on DSSQ worry, ECG IBI, or fNIR.

**Table 1:** ANOVA summary statistics for stress response measures that show significant stressor effects.

Measure	Stressor level		Stressor type		Stressor level × stressor type	
	*F*	$\eta_{p}^{2}$	*F*	$\eta_{p}^{2}$	*F*	$\eta_{p}^{2}$
**Subjective scales**						
Task engagement	1.35	0.020	3.48	0.049	16.96^∗∗^	0.202
Distress	34.46^∗∗^	0.340	80.33^∗∗^	0.545	28.10^∗∗^	0.295
Workload	27.95^∗∗^	0.294	93.51^∗∗^	0.583	28.18^∗∗^	0.296
**Physiological measures**						
ECG: HRV	12.90^∗∗^	0.163	1.29	0.019	0.82	0.012
TCD: CBFV^1^	6.65^∗∗^	0.097	0.90	0.014	5.51^∗^	0.082
EEG: Theta	5.76^∗^	0.081	0.53	0.008	1.82	0.027
EEG: Alpha	4.69^∗^	0.067	1.49	0.022	3.82	0.056
EEG: Beta	16.55^∗∗^	0.203	0.08	0.001	1.04	0.016
EEG: Gamma	18.44^∗∗^	0.221	1.41	0.021	0.31	0.005
**Performance measures**						
Command ratio	78.46^∗∗^	0.539	97.32^∗∗^	0.592	85.49^∗∗^	0.561
Search accuracy	6.41^∗^	0.087	1.54	0.023	0.13	0.002
Waypoints added	12.02^∗∗^	0.152	5.89^∗^	0.081	8.75^∗∗^	0.116

^∗^*p < 0.05, ^∗∗^*p* < 0.01. ^*1*^Analysis also included hemisphere factor*.

**Figure 2:**
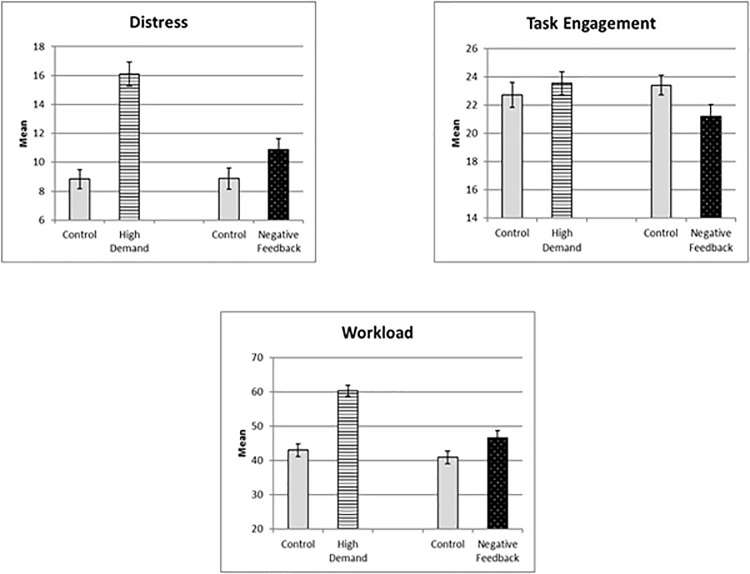
Stressor effects on three subjective state and workload measures.

Figure 2 illustrates stressor effects on subjective variables. Both stressors increased distress and workload, but both effects were stronger for the cognitive demand manipulation, as evidenced by the significant interactions between factors. For task engagement, only the interaction reached significance. Cognitive demand increased engagement slightly, whereas negative feedback reduced engagement more substantially.

**Figure 3:**
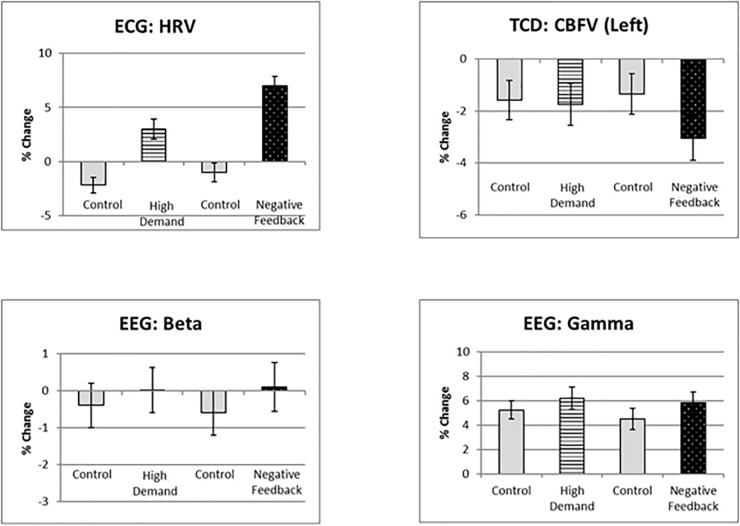
Stressor effects on four psychophysiological response measures.

Figure 3 shows principal stressor effects on the physiological variables. For most, only the main effect of stress level was significant. Both manipulations tended to increase heart rate variability and high frequency EEG spectral power (beta and gamma). Small-magnitude increases in theta and alpha under stress (not graphed) were also obtained. The only stressor-specific effect was for CBFV; blood flow velocity was lowest in the negative feedback condition.

A similar analysis of performance measures showed significant stressor effects on all three performance measures. The command ratio was lower in the high demand condition (*M* = 0.57, *SD* = 0.10) compared to the negative feedback condition (*M* = 0.77, *SD* = 0.12), the control condition for high demand (*M* = 0.77, *SD* = 0.11), and the control condition for negative feedback (*M* = 0.70, *SD* = 0.08). Search accuracy (proportion correct) was lower in both the high demand condition (*M* = 0.84, *SD* = 0.10) and in the negative feedback condition (*M* = 0.85, *SD* = 0.07) relative to the respective control conditions (*M* = 0.85, *SD* = 0.09; *M* = 0.87, *SD* = 0.08). The number of waypoints set was higher in the high demand condition (*M* = 4.54, *SD* = 3.49) than in the negative feedback condition (*M* = 3.19, *SD* = 2.50), or in the two respective control conditions (*M* = 2.91, *SD* = 2.47; *M* = 3.06, *SD* = 2.90). This last effect primarily reflects the need to set more waypoints when there are larger number of vehicles to direct.

### Associations Between Traits and Stress States: Baseline and Control Conditions

Table 2 shows intercorrelations of the traits and subjective state measures at pre-task baseline. All AnTI scales were associated with higher DSSQ worry, and also with lower task engagement, showing relationships with anticipatory stress. The AnTI, especially its social worry and meta-worry scales, was significantly negatively correlated with both hardiness and grit scales, with the exception of the challenge subscale. Hardiness and grit were correlated with more positive subjective states, but, by contrast with the AnTI, they were associated with (lower) distress, rather than with worry.

**Table 2:** Correlations between resilience traits and DSSQ state measures at baseline and in control conditions.

Scale	Measure	Mean (SD)	1	2	3	4	5	6	7	8	9
AnTI	1. Total	22.1 (3.4)									
	2. Social	18.4 (5.1)	0.901^∗∗^								
	3. Health	9.4 (3.4)	0.782^∗∗^	0.523^∗∗^							
	4. Meta-worry	12.8 (4.1)	0.900^∗∗^	0.724^∗∗^	0.606^∗∗^						
Hardiness	5. Total	74.6 (7.9)	-0.321^∗∗^	-0.344^∗∗^	-0.140	-0.311^∗∗^					
	6. Commitment	27.0 (3.8)	-0.383^∗∗^	-0.376^∗∗^	-0.261^∗^	-0.337^∗∗^	0.843^∗∗^				
	7. Control	26.1 (3.4)	-0.263^∗^	-0.292^∗^	-0.123	-0.236	0.757^∗∗^	0.552^∗∗^			
	8. Challenge	21.4 (3.4)	-0.054	-0.088	0.092	-0.111	0.629^∗∗^	0.290^∗^	0.144		
Grit	9. Total	3.5 (0.5)	-0.406^∗∗^	-0.356^∗∗^	-0.337^∗∗^	-0.361^∗∗^	0.271^∗^	0.349^∗∗^	0.318^∗∗^	-0.078	
DSSQ (pre-task)	10. Engagement	22.2 (5.6)	-0.298^∗^	-0.258^∗^	-0.276^∗^	-0.245^∗^	0.387^∗∗^	0.508^∗∗^	0.273^∗^	0.058	0.352^∗∗^
	11. Distress	9.5 (5.5)	0.177	0.286^∗^	-0.006	0.121	-0.333^∗∗^	-0.317^∗∗^	-0.300^∗^	-0.120	-0.318^∗∗^
	12. Worry	13.7 (5.6)	0.439^∗∗^	0.411^∗∗^	0.390^∗∗^	0.335^∗∗^	-0.017	-0.111	0.032	0.052	-0.138
DSSQ (control conditions)	10. Engagement	23.1 (5.6)	-0.192	-0.167	-0.232	-0.113	0.257^∗^	0.460^∗∗^	0.043	0.040	0.210
	11. Distress	8.8 (5.3)	0.194	0.256^∗^	0.033	0.171	-0.250^∗^	-0.304^∗^	-0.125	-0.115	-0.270^∗^
	12. Worry	6.0 (5.0)	0.330^∗∗^	0.335^∗∗^	0.198	0.299^∗^	-0.287^∗^	-0.323^∗∗^	-0.148	-0.158	-0.272^∗^

^∗^*p < 0.05, ^∗∗^*p* < 0.01*.

Subjective state variables were averaged across the two control conditions, to estimate state when task demands were undemanding. The correlations across the two control conditions for the DSSQ scales were 0.53 (task engagement), 0.66 (distress), and 0.73 (worry), showing individual differences were fairly consistent across the two conditions. The AnTI scales (except health worry) remained significantly positively correlated with state worry, but associations with task engagement were non-significant. DSSQ correlates of grit and hardiness were similar to those at baseline, with some differences in detail; for example, in the control conditions, both traits were significantly negatively correlated with state worry. Correlations between the trait scales and psychophysiological measures in the control conditions were also calculated but significant associations were few, and did not suggest any clear relationship between the traits and stress responses (data are available from the authors on request).

### Worry and Resilience Traits and Reactivity to Stressors

We calculated residualized indices of reactivity by regressing each subjective and physiological stress response measure for the two stressor conditions against the same measure in the matched control condition. For example, state worry for the negative feedback condition was regressed against state worry in the preceding control condition, and the standardized residual was calculated. The residual expresses the extent to which the measure is higher or lower than its value in the control condition predicts. Cross-stressor correlations in residuals were all non-significant, e.g., the three DSSQ residual correlations ranged from 0.08 to 0.18.

Table 3 shows correlations between the trait measures and residuals for the subjective state variables, for negative feedback and cognitive demand stressors. The AnTI showed a highly specific set of associations with worry reactivity. Total AnTI score, and two out of three subscales, were significantly correlated with state worry response. The additional resilience traits were more broadly correlated with reactivity. Total hardiness was associated with an attenuated distress response to both stressors, and with reduced worry in the negative feedback condition. All three hardiness subscales predicted lower distress response to negative feedback. Grit was exclusively associated with reactivity to the cognitive demand stressor, specifically with higher task engagement and lower distress.

**Table 3:** Correlations between resilience trait measures and stress reactivity: Subjective response (residualized).

		Negative feedback			Cognitive demand		
Scale	Measure	Engagement	Distress	Worry	Engagement	Distress	Worry
AnTI	Total	0.056	0.118	0.284^∗^	0.161	0.151	0.106
	Social	0.048	0.101	0.261^∗^	0.212	0.203	0.141
	Health	-0.030	0.103	0.285^∗^	0.172	-0.008	0.075
	Meta-worry	0.113	0.105	0.196	0.022	0.156	0.046
Hardiness	Total	0.052	-0.394^∗∗^	-0.344^∗∗^	-0.221	-0.247^∗^	0.088
	Commitment	0.122	-0.306^∗^	-0.212	0.170	-0.143	-0.050
	Control	0.114	-0.246^∗^	-0.198	0.064	0.014	-0.155
	Challenge	0.149	-0.282^∗^	-0.306^∗^	0.045	-0.004	-0.097
Grit	Total	0.009	-0.155	0.033	0.280^∗^	-0.345^∗∗^	0.157

^∗^*p < 0.05, ^∗∗^*p* < 0.01*.

Comparable correlations for residuals for selected psychophysiological measures are provided in Table 4. In this analysis, most of the correlations were non-significant, and the trait scales were significantly correlated only with EEG measures. Multiple significant correlates of theta and gamma response were found. The AnTI scales were associated with weaker theta and stronger gamma response. The hardiness commitment scale along with grit predicted stronger theta response; commitment also predicted lower gamma.

**Table 4:** Correlations between resilience trait measures and stress reactivity: EEG response (residualized).

		Negative feedback				Cognitive demand			
Scale	Measure	Theta	Alpha	Beta	Gamma	Theta	Alpha	Beta	Gamma
AnTI	Total	-0.323**	-0.004	0.250*	0.342**	-0.133	-0.241*	-0.107	-0.092
	Social	-0.289*	-0.076	0.217	0.342**	-0.097	-0.204	-0.128	-0.107
	Health	-0.324**	0.009	0.209	0.258*	-0.223	-0.224	-0.043	-0.031
	Meta-worry	-0.235	0.077	0.227	0.274*	-0.050	-0.205	-0.092	-0.087
Hardiness	Total	0.119	-0.021	-0.169	-0.187	0.082	0.144	0.001	0.005
	Commitment	0.252*	0.077	-0.161	-0.255*	0.035	0.101	0.048	0.051
	Control	0.065	-0.031	-0.119	-0.133	0.015	0.025	-0.033	0.013
	Challenge	-0.071	-0.105	-0.094	-0.015	0.137	0.199	-0.020	-0.059
Grit	Total	0.138	0.027	-0.094	-0.279*	0.147	0.085	-0.002	0.035

^∗^*p < 0.05, ^∗∗^*p* < 0.01*.

### Metacognition and the Functional Effects of Worry

It was hypothesized that individuals high in AnTI meta-worry would be more likely to show maladaptive responses with increasing state worry, relative to those low in meta-worry. Given the theoretical rationale for meta-worry being more likely to influence stress response to negative feedback than to cognitive demand, along with the preceding analyses, this hypothesis was tested only in the negative feedback condition, using a regression approach. Each performance and psychophysiological variable was treated as the dependent variable in turn.

**Figure 4:**
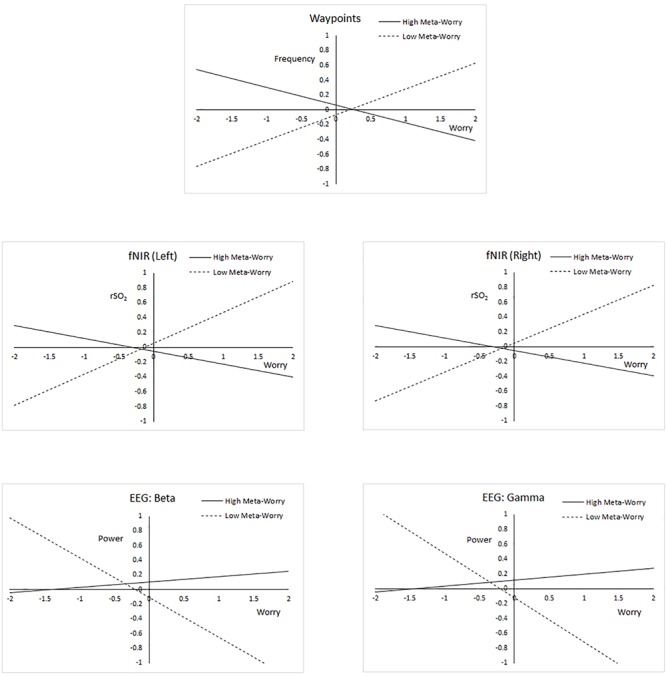
Regression plots illustrating interactions between meta-worry and state worry, for five outcome variables.

The dependent variable was predicted from linear terms for AnTI meta-worry and DSSQ state worry in the negative feedback condition, and the centered product term representing the interaction. In the analyses of performance, there were no significant linear or interactive effects for the command ratio or search accuracy measures. However, for waypoints added, the interaction was significant (β = -0.293, *p* < 0.05), though not the linear terms. The regression lines for individuals 1 SD above and below the mean are plotted in Figure 4 (top). As worry increases, individuals high in meta-worry assign progressively fewer waypoints, suggesting reducing task effort. Low meta-worry persons show the opposite trend.

For the physiological variables, the meta-worry × state worry interaction was significant for the left hemisphere fNIR rSO_2_ response (β = -0.296, *p* < 0.05), right fNIR rSO_2_ response (β = -0.279, *p* < 0.05), EEG beta (β = -0.308, *p* < 0.05), and EEG gamma (β = -0.338, *p* < 0.01). Linear terms were non-significant in all cases. The interactions for fNIR resemble those for waypoints added (Figure 4, center). Increasing worry appears to decrease frontal oxygen saturation in those high in meta-worry, with the opposite effect in low meta-worry individuals. Plots of the regression lines for high frequency EEG (beta and gamma) show that power tended to decrease with increasing worry in low meta-worry persons, with those high in meta-worry showing the opposite trend. These regressions include a linear trend toward decreasing power as state worry increases (significant at 0.05<*p* < 0.10 in both equations).

## Discussion

Traits for resilience predicted subjective and physiological responses to negative feedback and cognitive demand stressors in a multi-UAS control simulation. As expected, worry traits, including meta-worry, were generally associated with higher levels of situational stress, whereas hardiness and grit appeared protective. The data also revealed more subtle relationships between traits and stress outcomes. As predicted, the AnTI was predictive of stressor reactivity primarily in the negative feedback condition, consistent with cognitive-attentional theory (Wells and Matthews, 2015). The moderator effect of meta-worry on relationships between subjective state worry and objective stress responses was also consistent with theory; worry appears to be especially maladaptive for those high in meta-worry. Hardiness and grit were negatively correlated with the AnTI worry scales: maladaptive metacognitive style may impair development of a resilient personality. Table 5 summarizes the evidence supporting each of the major hypotheses of the study. The remainder of this discussion addresses the four relevant research questions, as well as limitations and practical applications of the study.

**Table 5:** Summary of major research questions and outcomes confirming hypotheses.

Research question	Hypothesis	Theory tested	Outcome
1. Stressor impacts	The two stressors will elicit overlapping but distinct patterns of response.	TSO	Confirmed. Both stressors elicited distress, high-frequency EEG, and increased HRV. Stressors were differentiated by effects on engagement and CBFV.
2. Predictors of baseline stress	Resilience traits will predict anticipatory stress.	CAS	Confirmed. All three resilience measures predicted pre-task subjective state.
3. Individual differences in stress reactivity	Traits will predict response to stressors, moderated by stressor and type of trait.	TSO, CAS	Confirmed. The three resilience measures predicted different patterns of stress response; e.g., AnTI predicted worry and EEG response to negative evaluation.
4. Functional impact of worry	State worry will have more harmful impacts in high meta-worry individuals.	CAS	Confirmed. State worry was associated with behavioral and physiological indicators of reduced effort in high meta-worry individuals.

### Stress Profiles of Cognitive Demand and Feedback Stressors

Both stressors elicited higher state distress, as expected, but the effect was larger for cognitive demand. High workload plays a major role in provoking the subjective distress response in task performance contexts, as the person appraises the task as uncontrollable and utilizes multiple forms of coping to manage overload (Matthews and Campbell, 2009; Matthews et al., 2013). Contrary to expectation, negative feedback did not elicit higher state worry. As discussed subsequently, the different stressors may have influenced the qualitative nature rather than the intensity of worry. The stressors were differentiated by task engagement, which declined under negative feedback, suggesting that it may have been demotivating. By contrast, task engagement was sustained in the high demand condition, consistent with evidence from other complex tasks that are sufficiently challenging to be motivating (Matthews, 2016).

Responses to stressors were less differentiated at the physiological level, with both eliciting increased power in high-frequency EEG bands. Both stressors also elevated HRV, a somewhat unexpected finding given that increased workload typically reduces this index. Phasic HRV increases may reflect emotion-regulation and successful engagement of cognitive inhibitory processes (Kemp et al., 2017). In the performance context, participants’ efforts to focus on a demanding though challenging task may have encouraged inhibitory strategies. The stressors were differentiated by the CBFV response, which was lower in the negative feedback condition. Declining CBFV is typically a marker for loss of sustained attention and vigilance (Warm et al., 2012); it corresponds to the loss of task engagement also seen in this stressor condition.

Overall, the findings suggest that both manipulations induced substantial subjective stress, but not the classical sympathetic arousal response, given that there was no stressor effect on mean heart rate. Instead, the marked increase in high-frequency EEG power suggests a more “cognitive” expression of stress that may reflect performance concerns and, as suggested by the HRV responses (Kemp et al., 2017), efforts at cognitive stress-regulation. From the military perspective, stress of this kind may become increasingly significant as Warfighters shift from active combat roles to those that are remote from physical danger such as controlling unmanned vehicles and cyber operations. The greater differentiation of stressor impacts in the subjective data supports previous findings that physiological and subjective indices reflect distinct elements of the stress response, both of which add to evaluation of operator functioning (Matthews et al., 2017b).

### Associations Between Traits and Stress States: Baseline and Control Conditions

Previous studies found that trait worry predicts a range of stress outcomes (e.g., Roussis and Wells, 2006; Spada et al., 2008, 2010). Analyses of state data from the baseline and control conditions, confirmed that the AnTI predicted higher worry, even in the absence of an overt stressor. Individuals high in trait worry and meta-worry may be prone to anticipate that the task will pose a threat (O’Carroll and Fisher, 2013), and to focus their attention on threat concerns even in undemanding task conditions (Spada et al., 2008). The AnTI also predicted lower baseline task engagement. Grit and hardiness scales were generally more predictive than the AnTI of distress, and of task engagement in control conditions. Consistent with the TSO framework (Matthews et al., 2017a), multiple trait measures are required to define the individual’s stress vulnerability.

Total scores on the hardiness and grit scales were both negatively associated with AnTI meta-worry. We cannot make casual inferences from cross-sectional data, but these associations are at least compatible with a role for dysfunctional metacognitions in undermining resilience. Hardiness and grit both support persistence in the face of adversity through active coping with obstacles to personal goals (Bartone et al., 1989; Duckworth and Quinn, 2009; Kelly et al., 2014). Effective coping may be more difficult if attention is directed toward self-referent worry and rumination (Hong, 2007). Indeed, a longitudinal study found that metacognitive style predicted subsequent anxiety, in a non-clinical sample (Ryum et al., 2017).

### Worry and Resilience Traits and Reactivity to Stressors

The study tested whether traits were associated with reactivity to the two stressors, over and above any general tendency toward higher levels of stress. Reactivity to stressors was assessed using residualized measures capturing the unique response to the stressor concerned. Consistent with the TSO framework (Matthews et al., 2017a), cross-stressor correlations for reactivity measures were close to zero, and associations between traits and reactivity varied with the trait and with the outcome measure. In fact, there was a double dissociation between worry traits and grit, with the AnTI predicting only reactivity to negative feedback, and grit predicting only reactivity to high demand. The AnTI was selectively related to worry response, consistent with the S-REF model (Wells and Matthews, 2015). Overall state worry levels in task conditions were quite low, due to substantial cognitive workload directing attention outward to task stimuli (Matthews et al., 2013). Nevertheless, individuals high in the various facets of trait worry assessed by the AnTI appeared to be sensitive to state worry. The nature of worries activated in the two stressor conditions may have differed, with negative feedback eliciting self-referent concerns about personal competence, and the high demand condition activating concerns more directly related to task goals.

Hardiness correlated with reactivity to both stressors, but it was generally more predictive of response to negative feedback than to cognitive demand. Total hardiness was associated with attenuated distress and worry responses to the feedback manipulation, the study stressor more likely to promote self-evaluation. Hardiness is associated with styles of appraisal and coping that are adaptive in a performance setting (Eschleman et al., 2010; Cash and Gardner, 2011; Escolas et al., 2013) and are likely to suppress the CAS (Wells and Matthews, 2015). The challenge component of hardiness was the best predictor of reduced worry response, the primary symptom of CAS suppression. Thus, the capacity to embrace uncertainty over personal competence and see it as a positive experience may be the element of hardiness that counteracts the tendency for negative feedback to elicit the CAS, and points toward a need to further investigate metacognitive aspects of the trait.

Grit predicted higher task engagement and lower distress under high demand; the motivational qualities associated with grit may be especially important under these circumstances. Task engagement is associated both with intrinsic motivation and striving for performance excellence (Matthews et al., 2001). The role of grit in promoting task engagement is consistent with Lucas et al.’s (2015) finding that high grit participants persist with a difficult task even when they are failing. Grit also correlated with positive emotion and expectancies under these circumstances. Here, the more adaptive subjective state response to high workload experienced by those high in grit may be a consequence of self-regulative processes such as maintaining a sense of self-efficacy (Muenks et al., 2018) and adaptive management of task demands (Wolters and Hussain, 2015) that are especially well-suited for dealing with cognitive overload.

The traits were more weakly associated with physiological measures of stressor reactivity than with the subjective ones, but the negative feedback EEG data were notable for the consistent set of associations between higher AnTI scores and lower theta and higher gamma response. Theta and gamma may be functionally inter-related, based on evidence for cross-phase coupling (Belluscio et al., 2012). Both frequency bands are influenced by emotion-regulation (Tolegenova et al., 2014), as well as by demanding cognitive processing (Ishii et al., 2014). A magnetoencephalography (MEG) study showed overlapping theta and gamma synchronization responses to emotional stimuli in multiple brain areas including amygdala and frontal cortex (Luo et al., 2014). Tentatively – and with due regard for the challenges of using EEG to infer brain processes – the data may signal individual differences in cognitive regulation of emotion. Higher gamma in higher-worry individuals is attributed to negative emotional arousal and anxiety (Headley and Paré, 2013), and disproportionate worrying (Oathes et al., 2008), whereas lower frontal theta indicates lower task-directed effort (Gevins and Smith, 2003), poorer working memory maintenance (Hsieh and Ranganath, 2014), and unsuccessful emotion-regulation (Ertl et al., 2013). Conversely, the high theta/low gamma pattern of the low AnTI scorer may reflect successful emotion-regulation that supports task-directed attention and mitigation of anxiety and worry. Frontal gamma desynchronization may also be associated with a mechanism for interrupting task-irrelevant cognitive activity (Ishii et al., 2014).

### Metacognition and the Functional Significance of Worry

Results thus far discussed suggest AnTI trait worry showed a distinctive pattern of associations with stress outcomes including generally higher state worry along with a more specific subjective and EEG response to negative feedback that may indicate poor emotion-regulation. However, these findings do not indicate a specific adaptive role for metacognition, i.e., meta-worry. The final set of analyses aimed to investigate the role of meta-worry in maladaptive stress outcomes by testing whether it moderated objective correlates of state worry.

A moderator effect of meta-worry was found for the number of waypoints used, but not for the two overall performance measures. Behaviorally, in high meta-worry persons, state worry appeared to reduce task-directed effort, i.e., setting simpler paths to avoid hazards. By contrast, those low in meta-worry seemed to try harder as they become more worried. For these individuals, the worry state may be adaptive in motivating adaptive and coping task effort, blocking development of the CAS (Wells and Matthews, 2015). However, in individuals with high meta-worry, which is a marker for negative beliefs about the uncontrollability and danger of the worry process (Wells, 2005), full CAS activation occurs as the individual diverts resources to mental self-regulation. Findings parallel Eysenck and Calvo’s (1992) proposal that anxious individuals preserve processing effectiveness through compensatory effort. Berggren and Derakshan’s (2013) review of the evidence for the hypothesis found mixed results. One explanation for inconsistency in findings is that the compensatory effort hypothesis is only valid for individuals low on dysfunctional metacognitions. Tentatively, compensatory effort might be impaired in high meta-worry because knowledge concerning control of attention is compromised and greater imminent threat is posed by cognition itself. A similar finding is evident in pathological worry, in which individuals with generalized anxiety disorder (GAD) report that worry is advantageous for coping and motivation, but it appears to become disruptive to functioning and be a characteristic feature of GAD when meta-worry develops (Wells and Carter, 2001).

The physiological findings are consistent with this explanation. fNIR measures are indicative of task workload (Ayaz et al., 2012). On this metric, high meta-worry individuals show declining workload as state worry increases, implying reduced on-task effort. The concurrent increases in high-frequency EEG, including gamma, may be associated with the activation of the CAS (Wells, 2009), and self-focused attention as the person attempts to process the significance of their own worries.

More generally, the findings suggest a re-evaluation of the functional significance of worry in performance environments. Typically, worry is seen as a detrimental influence, as in classic studies of cognitive interference and test anxiety (Sarason et al., 1995). However, meta-analyses of the association between worry and measures of academic performance suggest that the effect size for the correlation is a modest -0.2 or so (e.g., Richardson et al., 2012). Studies of various attentional tasks have suggested that worry is typically a weaker correlate of poor performance than low task engagement and/or high stress (Matthews, 2016). The present findings support more nuanced accounts of worry (e.g., Sweeny and Dooley, 2017) that identify possible motivational benefits to the state. Similarly, studies of stress and skilled performance suggest some individuals are able to utilize stress symptoms as a motivator, for example, in sports (Matthews et al., in press).

The current study identifies metacognition as a critical determinant of the consequences of worry. A somewhat comparable moderator effect was obtained by Nordahl and Wells (2017), in a sample of socially anxious individuals. Metacognitive belief was the only one of several cognitive variables that uniquely predicted whether the person was working or not. Dysfunctional metacognitions may limit the person’s capacity to function despite social anxiety. A similar study with a more diverse sample showed that metacognitive beliefs about the need for mental control predicted whether the person was working or on disability benefits, over and above trait anxiety and mental disorder (Nordahl and Wells, 2018). Dysfunctional metacognitions may limit the person’s capacity to function despite social anxiety and other emotional conditions.

The present findings support the central proposition of S-REF theory that meta-cognitive dysfunction is a major driver of worry states (Wells and Matthews, 2015). Maladaptive beliefs about worries are an element of stable self-knowledge associated with personality that increases the likelihood of CAS activation. Metacognitions refer to both beliefs about processes, such as the importance of attending to intrusive thoughts, and to specific beliefs about thought contents (Wells, 1995), In challenging performance contexts, worry may indeed be elevated by process-based metacognitions, consistent with test anxiety research (Matthews et al., 1999). However, the distinctiveness of worry and metacognition as constructs is confirmed by the finding that the objective correlates of state worry vary with metacognitive style. The role of thought content in the performance context merits further investigation: for example, high meta-worry individuals may interpret thoughts of failure as actual failure.

### Limitations

The current study used a student sample asked to perform a complex task simulation following a relatively short training and practice period. Generalization of findings to samples of expert UAS operators is thus questionable. Greater skill and experience may attenuate stress response (Matthews et al., in press), but there is also more at stake in the real environment, which might elevate stress. Furthermore, operators face chronic stressors such as long work-shifts (Tvaryanas et al., 2006) that may moderate acute response. Lack of experience with the task may also have limited the validity of the performance measures. We observed substantial performance variability across participants; longer test sessions that allowed participants to develop a stable performance strategy would have been desirable. From a clinical perspective, relationships between personality and stress variables found in non-clinical samples will not necessarily generalize to clinical populations, given that relatively mild stress states may not represent severe clinical anxiety conditions well.

There are also issues related to stress assessment. To keep the data analysis tractable, we calculated responses averaged across each task condition, but there may have been considerable variation in stress within each condition. Further research might test the role of metacognitive style in response to discrete, high-stress events. The experiment was also not designed to investigate dynamic stress processes, such as changes in coping strategy within experimental conditions. The study exemplifies a multivariate assessment approach that specifies a profile of subjective and objective stress response across multiple measures (Matthews, 2016; Matthews and Reinerman-Jones, 2017). The differing sensitivities of the various measures justify the multivariate approach, but its application also multiplies the number of analyses and the risk of chance findings. The current study aimed to guard against this danger by using theory to guide data analysis, but replication of findings would be desirable. Conversely, more advanced analytic techniques could refine measures, such as spectral frequency analysis of the ECG to better separate sympathetic and parasympathetic response components (e.g., Kemp et al., 2017). On the predictor side, the AnTI meta-worry scale assesses only a single aspect of metacognitive style, and there are further dimensions of metacognition that may moderate stress response (e.g., Wells and Cartwright-Hatton, 2004; Wells, 2005).

## Conclusion

The current study confirms that traits for worry, hardiness and grit predict stress response in a complex multi-UAS control environment. Findings support the central tenet of the TSO framework (Matthews et al., 2017a) that resilience is a multifaceted construct. The predictive validity of resilience and stress-vulnerability traits varied across stressors and across stress outcome measures. Within this broad framework, the role of the AnTI worry traits in predicting outcomes was consistent with the S-REF model (Wells and Matthews, 2015). Worry traits were more relevant to negative feedback than to cognitive demand, and they appeared primarily to influence state worry and EEG bands that may reflect attempts at emotion-regulation. The S-REF model also predicted that the functional significance of worry states would vary with metacognition (meta-worry). Findings within the negative feedback stressor condition suggested that the maladaptive CAS may accompany worry states only when the person is disposed to dysfunctional metacognitions.

From an applied standpoint, the data support multifactorial assessments of populations required to perform complex or otherwise stressful tasks, including military populations. The various stressors prevalent in the UAS environment (Tvaryanas et al., 2006) may elicit qualitatively different stress responses, requiring different strategies for mitigation. Teaming situations in particular may involve negative evaluation from team-mates, especially when inexperienced teams are required to tackle difficult tasks that strain team cohesion. Current personnel selection emphasizes broad measures of negative affectivity such as neuroticism in the Five Factor Model (Huang et al., 2014), but more narrowly specified traits, including those for metacognitive dispositions, may improve predictive validity for performance under stress, especially if the trait can be matched to the stressor appropriately.

Profiling strengths and vulnerabilities may also allow training to be tailored to the individual to optimize resilience. For example, Wells (2000) Attention Training Technique (ATT) is a component of metacognitive therapy that is also effective for mitigating anxiety in non-clinical samples (Fergus and Wheless, 2018). ATT might help operators high in meta-worry manage evaluative stress. By contrast, interventions designed to enhance task motivation or strategy might be better suited to help operators lacking grit deal with high workloads.

## Ethics Statement

This study was carried out in accordance with the recommendations of the University of Central Florida Internal Review Board; with written informed consent from all subjects. All subjects gave written informed consent in accordance with the Declaration of Helsinki. The protocol was approved by the University of Central Florida Internal Review Board.

## Author Contributions

All authors listed have made a substantial, direct and intellectual contribution to the work, and approved it for publication.

## Conflict of Interest Statement

The authors declare that the research was conducted in the absence of any commercial or financial relationships that could be construed as a potential conflict of interest.
